# Aesthetical and Accuracy Outcomes of Reconstruction of Maxillary Defect by 3D Virtual Surgical Planning

**DOI:** 10.3389/fonc.2021.718946

**Published:** 2021-10-19

**Authors:** Yang Wang, Xingzhou Qu, Junjian Jiang, Jian Sun, Chenping Zhang, Yue He

**Affiliations:** ^1^ Department of Oral & Maxillofacial–Head & Neck Oncology, Shanghai Ninth People’s Hospital, Shanghai Jiaotong University School of Medicine, Shanghai, China; ^2^ National Clinical Research Center for Oral Diseases, Shanghai Key Laboratory of Stomatology & Shanghai Research Institute of Stomatology, Shanghai, China; ^3^ Shanghai Key Laboratory of Stomatology, Shanghai Research Institute of Stomatology, Shanghai, China

**Keywords:** maxillary defect, virtual surgical planning (VSP), 3D printing, free flap, accuracy reconstruction

## Abstract

**Background:**

Reconstruction of maxillary defect resulting from trauma or oncology surgery is of great importance for patients with physical and psychological complications. The virtual surgical planning (VSP) and 3D printing technics had been used in recent years which simplified the surgical procedure and promoted success and accuracy. To assess the accuracy and outcome of VSP surgery, here we report our experience in maxillary reconstruction retrospectively.

**Method:**

Patients who received maxillary defect reconstruction from 2013 to 2020 were analyzed retrospectively. These patients were divided into two groups. Group 1 received VSP and 3D printed guiding plates in the surgery, while group 2 underwent free-hand surgery (FHS). Patients with different vertical and horizontal defects were classified according to Brown and Shaw classification. Clinical information and postoperative complications of all patients were collected. For patients with unilateral maxillary defect, orbit volume, orbit height, and the contour of the reconstructed side were compared with the normal side.

**Result:**

Thirty-four patients who achieved the criteria were analyzed, of which 20 patients underwent VSP surgery. There were primary and secondary reconstruction cases in both two groups. Vascularized iliac crest flap was used in three cases, and fibula flap was performed in the other cases. One flap collapse occurred in FHS group. Seven patients in VSP group received dental implants, while the number in FHS group was 0. In vertical class III cases, the differences in orbit height (ΔD) and orbit volume (ΔV) between normal side and reconstructed side were measured and compared in the two groups. The mean ΔD is 1.78 ± 1.33 mm in VSP group and 4.25 ± 0.95 mm in FHS group, while the mean ΔV is 2.04 ± 0.85 cm^3^ in VSP group and 3.25 ± 0.17 cm^3^ in FHS group. The alterations of orbit height and volume in VSP group were much smaller than that in FHS group with statistical significance. From the perspective of aesthetics, the color-gradient map indicates a more symmetric and smoother curve of post-operation appearance in VSP group.

**Conclusion:**

Compared with traditional free-hand surgical technics, VSP and 3D printing guiding plates can allow for a more accurate maxillary reconstruction with improved aesthetics.

## Introduction

The reconstruction of maxilla or midface with the rehabilitation of occlusion, speech, and normal vision was challenging in head and neck reconstructive surgery. Patients with maxillary defects usually suffered great psychological and physical trauma, compelling surgeons to try their best to achieve the ideal result of maxillary reconstruction. The optimal maxillary reconstruction includes separation of oral cavity and nasal cavity, rehabilitation of alveolar ridge and maxillary buttress, restoration of normal visual function, and satisfied soft tissue contour (1–3). With the development of microvascular surgery, several free flaps had been described for use in reconstruction of the maxillary defect, such as anterolateral thigh flap, scapular flap, fibula osteomyocutaneous flap, iliac crest flap, et al. (2–10).

In the past two decades, virtual surgical planning (VSP) had been developed to improve the surgical and anatomic accuracy, and consequently aesthetical appearance, vision function, and occlusion. Due to the high accuracy of VSP, surgical resections with good tumor margin control can be obtained during ablation (11–13), and the use of bone flap containing several segments can be possible and simplified. To assess the accuracy and outcome of VSP surgery, here we report our experience in maxillary reconstruction retrospectively, including traditional free-hand surgery (FHS) and VSP surgery. The facial appearance and reconstruction outcome of both groups were evaluated. The VSP surgery group exhibited better accuracy and improved satisfaction with appearance. Still there are some special considerations that need to be addressed.

## Patient and Methods

### Patients

From 2013 December to 2020 October, 34 patients undergoing maxillary reconstruction in Department of Oral & Maxillofacial–Head & Neck Oncology, Shanghai Ninth People’s Hospital were incorporated, including 20 patients receiving operation with the use of VSP and three-dimension (3D) printing technics, and 14 patients received FHS. Clinical details are shown in **Table 1** and **Supplementary Table S1**. The maxillary vertical and horizontal defects were classified according to Brown’s revised defect classification of maxilla and midface (14). Primary diagnosis result in the defect included malignant tumor, benign tumor, and trauma. Surgical techniques including donor site of flap, usage of titanium mesh or Polyetheretherketone (PEEK), and recipient artery were recorded. Complications and the treatment during follow-up were traced. Our study was approved by the Ethics Committee of Shanghai Ninth People’s Hospital, Shanghai Jiao Tong University School of Medicine.

**Table 1:** Patient demography and clinical details.

Patient demography	VSP(N)	FHS(N)
**Gender**		
Male	14	9
Female	6	5
**Age at surgery**		
<20	3	0
20–40	11	8
40–60	6	6
**Maxilla defect reason**		
Malignant tumor	13	8
Benign tumor	6	5
Trauma	1	1
**Stage**		
Primary reconstruction	7	7
Secondary reconstruction	13	7
**History of RT at surgery**		
Yes	8	3
No	12	11
**Brown’s classification**		
**Vertical defect**		
Class II	12	9
Class III	8	5
**Horizontal defect**		
Class b	12	9
Class c	2	2
Class d	6	3
**Reconstructive technique**		
Fibula free flap	17	14
Segments 1	0	1
2	9	3
3	7	9
4	1	1
Vascularized iliac bone flap	3	0
Titanium mesh	4	3
PEEK	1	0
**Recipient artery**		
Superfacial temporal artery	16	7
Facial artery	3	6
Superior thyroid artery	1	1
**Dental implant**		
Yes	7 (35%)	0
No	13	14
**Surgery complications**		
Flap vascular crisis	3 (15%)	2 (14.3%)
Flap failure	0	1 (7.14%)
Inflammation	1 (5%)	2 (14.3%)
Exposure of titanium mesh or PEEK	3 (15%)	2 (14.3%)
Enophthalmos	3 (15%)	3 (21.4%)

### Virtual Surgical Planning and Surgical Techniques

**Figure 1:**
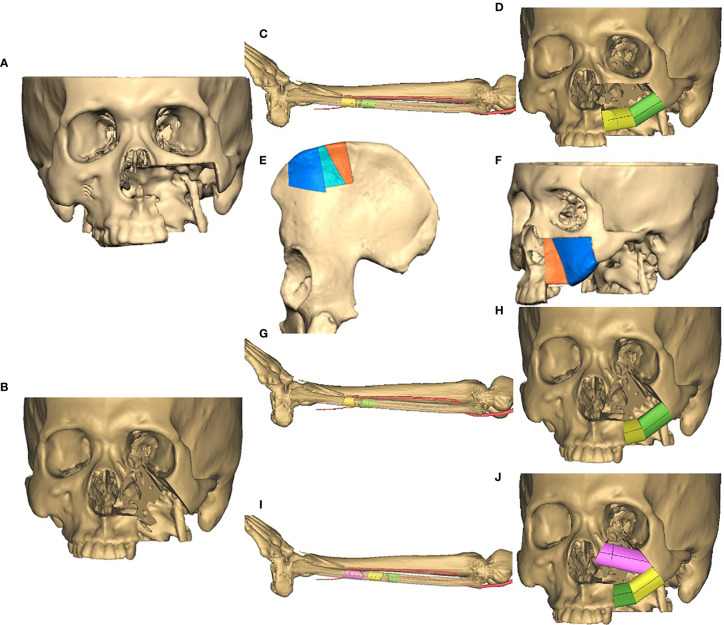
Virtual surgical planning (VSP) strategy. **(A)** A virtual maxilla vertical defect of Class II; **(B)** a virtual maxilla vertical defect of Class III; **(C)** a virtual plan of osteotomy on fibula for defect in **(A)**, two segments were designed to render the curve of alveolar ridge and pterygomaxillary buttress; **(D)** virtual position of fibular segments according to defects in **(A, C)**; **(E)** a virtual plan of osteotomy on iliac crest for defect in **(A)**, osteotomies were performed to generate three segments, and the middle one was removed; **(F)** virtual position of ICFF segments according to defects in **(A, E)**; **(G)** a virtual plan of osteotomy on fibula for defect in **(B)**, two segments were designed to render the curve of alveolar ridge and pterygomaxillary buttress; **(H)** virtual position of fibular segments according to defects in **(B, G)**, titanium mesh was used to restore the infraorbital rim; **(I)** a virtual plan of osteotomy on fibula for defect in **(B)**, three segments were designed to render the curve of infraorbital rim, alveolar ridge and pterygomaxillary buttress; **(J)** virtual position of fibular segments according to defects in **(B, I)**.

All patients underwent preoperative computed tomography (CT) scans of craniofacial and lower extremities or pelvis. These CT images were converted into 3D virtual models, and surgical planning was performed using Mimics software (Materialise, Leuven, Belgium). The 3D image of fibula or iliac crest free flap (ICFF) were superimposed onto the mirrored maxilla image reconstructed by the software, and osteotomies were performed in the digital fibula or ICFF so that the curve of the segments would match the contour of maxillary alveolar ridge and pterygomaxillary buttress (**Figure 1**).

**Figure 2:**
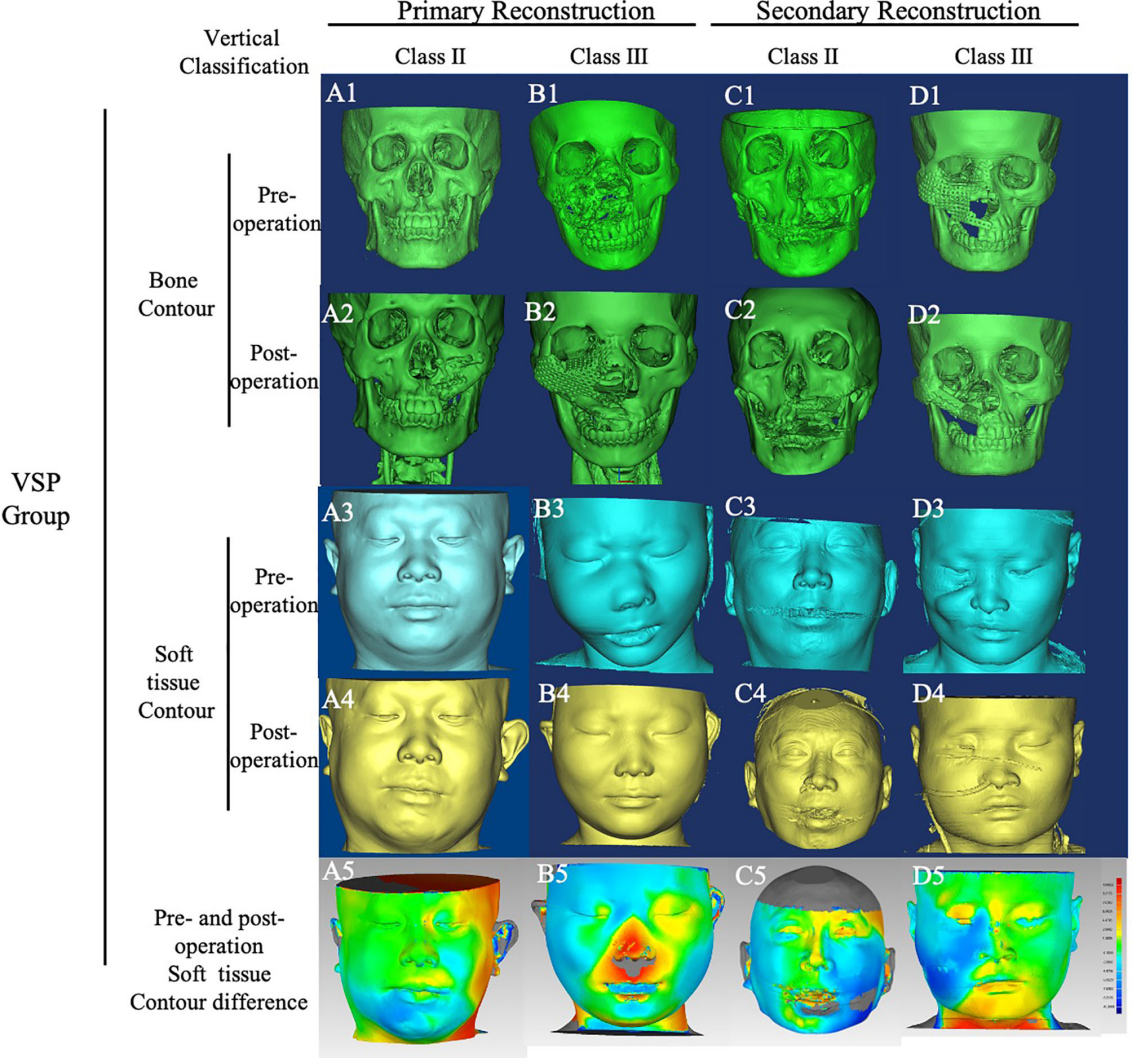
Bone and soft tissue contour of cases in VSP group. Cases in VSP group were classified by vertical defect classification and surgery stage. CT images of all cases were converted into Mimics, and the bone and soft tissue contours were calculated. The color-gradient map indicating differences between mirrored symmetrical face and postoperative soft tissue contour was generated by Geomagic studio. Red color indicates ridge area, and blue indicates depressed area. **(A)**, case of primary reconstruction with vertical defect class II; **(B)**, case of primary reconstruction with vertical defect class III; **(C)**, case of secondary reconstruction with vertical defect class II; **(D)** case of secondary reconstruction with vertical defect class III. A1~A5, B1~B5, C1~C5, D1~D5 showed the pre-operation bone contour, post -operation bone contour, pre-operation soft tissue contour, post-operation soft tissue contour, pre-operation and post-operation soft tissue contour difference respectively.

Fibula flap was used in most cases for maxillary reconstruction. For Brown’s class II vertical defects (**Figure 1D** and **Figure 2C**), the distal end of fibula bone graft was placed to the alveolar ridge according to the digital cutting plane, and the proximal end to pterygomaxillary cutting plane. So the vessel pedicle of fibula flap can traverse to anterior tragus area, and the anastomosis was accomplished with superficial temporal artery and vein. The reconstructed maxillary alveolar ridge was usually rehabilitated to the location of first molar, and another segment turned to form pterygomaxillary buttress in necessity.

For Brown’s class III vertical defects, there were two strategies. One design strategy was just like what was used for class II defects (**Figures 1G, H**). Additionally, titanium mesh was used to form the floor of orbit and the infraorbital rim. Individualized titanium mesh was prebended according to the 3D model of reconstructed maxilla. Soft tissue like fat tissue of the skin paddle or part of flexor pollicis longus muscle should be placed around the titanium mesh to prevent its exposure. In terms of the other strategy (**Figures 1I, J**), the distal end of fibula bone graft was placed to form infraorbital margin, and the proximal part was shaped to form the alveolar ridge. In this case, the digital cutting plane of alveolar ridge was restricted posterior to canine; therefore, the vessel pedicle can traverse to submandibular area through a tunnel in buccal, and anastomosis was accomplished with facial artery and vein or others.

For cases using ICFF (**Figures 1E, F**), the surgical planning was performed as described before (15). Briefly, the contour of maxilla could be divided into two subunits of paranasal region and infraorbital region through a line segment from nasion to distal alveolar crest of maxillary second premolar. Osteotomies were performed in the bone graft of ICFF, and three segments were generated. The middle segment was removed, and the other two segments were spliced to match the curve of maxillary alveolar ridge and pterygomaxillary buttress. For Brown’s class III vertical defects, the floor of orbit and the infraorbital rim were established by bone graft of ICFF, too.

### Special Surgical Technique

**Figure 3:**
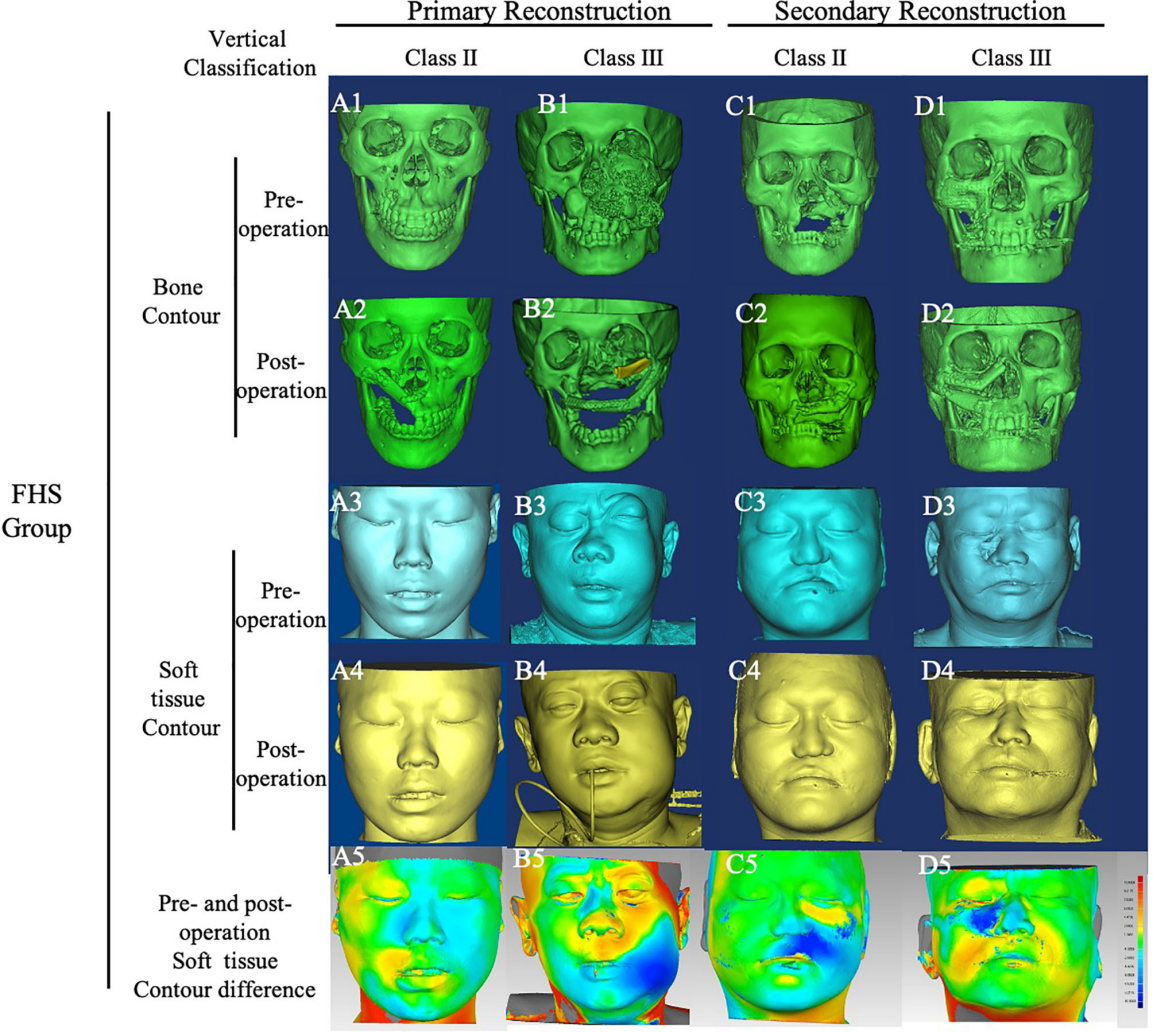
Bone and soft tissue contour of cases in FHS group. Cases in FHS group were classified by vertical defect classification and surgery stage. CT images of all cases were converted into Mimics, and the bone and soft tissue contours were calculated. The color-gradient map indicating differences between mirrored symmetrical face and postoperative soft tissue contour was generated by Geomagic studio. Red color indicates ridge area, and blue indicates depressed area. **(A)**, case of primary reconstruction with vertical defect class II; **(B)**, case of primary reconstruction with vertical defect class III; **(C)**, case of secondary reconstruction with vertical defect class II; **(D)** case of secondary reconstruction with vertical defect class III. A1~A5, B1~B5, C1~C5, D1~D5 showed the pre-operation bone contour, post -operation bone contour, pre-operation soft tissue contour, post-operation soft tissue contour, pre-operation and post-operation soft tissue contour difference respectively.

In FHS group, one case was graded class IIId (**Figure 3B**). To prevent enophthalmos, the anterior part of mandibular ramus including coracoid was cut off from sigmoid notch to retromolar area, with attachment of temporalis muscle. This pedicled bone graft was shaped and placed to form the floor of orbit (**Figure 3B2**). This technique was called coracoid-temporalis flap.

### Reconstructive Accuracy Analysis

**Figure 4:**
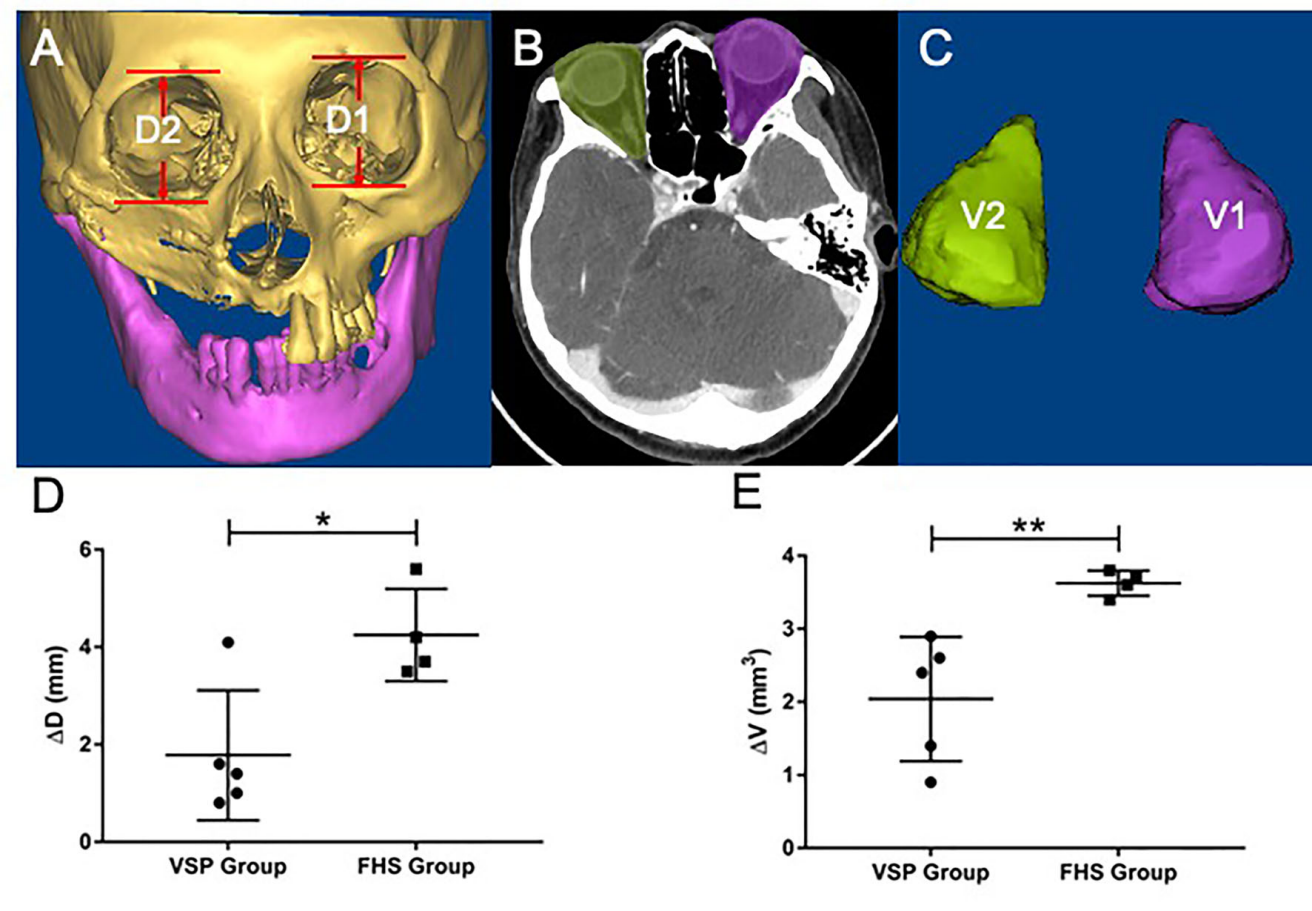
Orbit height and orbital volume analysis in VSP and FHS group. **(A)** The definition of D1 and D2. In the frontier view, two lines were drawn. The upper line was horizontal to the lowest point of supraorbital margin, and the lower line was horizontal to the highest point of infraorbital margin. In the normal side, the vertical distance between these two lines was defined as D1. In the reconstructed side, the vertical distance was defined as D2; **(B)** the content of orbit was delineated in the normal side and reconstructed side; **(C)** the 3D model of orbital volume was established, the volume of normal orbit was defined as V1, and the volume of reconstructed orbit was defined as V2, which was calculated by Materialise Magics; **(D)** the absolute value of difference between (D1, D2) was defined as ΔD, which was analyzed in both VSP and FHS groups by two-tailed t test with statistical significance (p<0.05); **(E)** the absolute value of difference between V1 and V2 was defined as ΔV, which was analyzed in both VSP and FHS groups by two-tailed t test with statistical significance (p<0.02). *p < 0.05; **p < 0.02.

For cases classified as Brown’s class III vertical defects, DICOM images of the postoperative CT scan were imported into Mimics software. Then a mask was established using bone tissue thresholds, and a 3D model was produced by calculation. In the frontier view, two lines were drawn as shown in **Figure 4A**. The upper line was horizontal to the lowest point of supraorbital margin, and the lower line was horizontal to the highest point of infraorbital margin. In the normal side, the vertical distance between these two lines was defined as D1. Accordingly, in the reconstructed side, the vertical distance was defined as D2. The absolute value of difference between D1 and D2 was defined as ΔD.

A new mask was established and the content of orbit was selected in all the slides (**Figure 4B**), then the 3D models of orbit were calculated (**Figure 4C**). The volume of the orbit 3D model was calculated using Materialise Magics software (Materialise, Leuven, Belgium). The volume of normal orbit was defined as V1, and the volume of reconstructed orbit was defined as V2. The absolute value of difference between V1 and V2 was defined as ΔV.

Both ΔD and ΔV were calculated in VSP group and FHS group. SPSS (Statistic Package for Social Sciences) 13.0 was used for data analysis. Two tailed t-test was used for statistical analysis.

**Figure 5:**
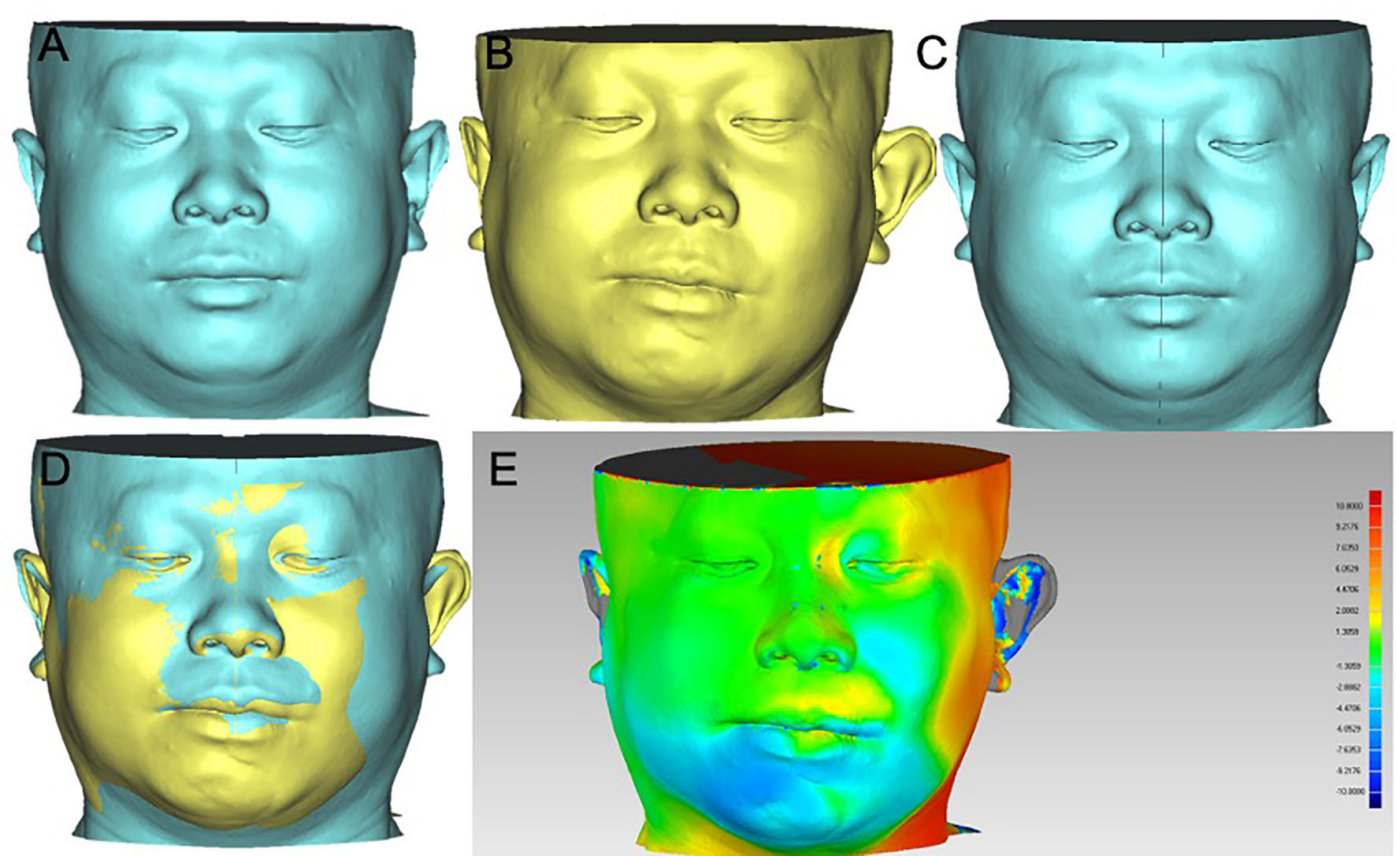
Soft tissue contour of pre-operation and post-operation alignment in VSP group. **(A)** Pre-operation soft tissue contour; **(B)** post-operation soft tissue contour; **(C)** mirrored hemiface of normal side to form a symmetrical face; **(D)** post-operation soft tissue contour aligned with the symmetrical face in **(C)**; **(E)** the color-gradient map indicating the differences of soft tissue contour in **(D)**, red color indicates ridge area, and blue indicates depressed area.

**Figure 6:**
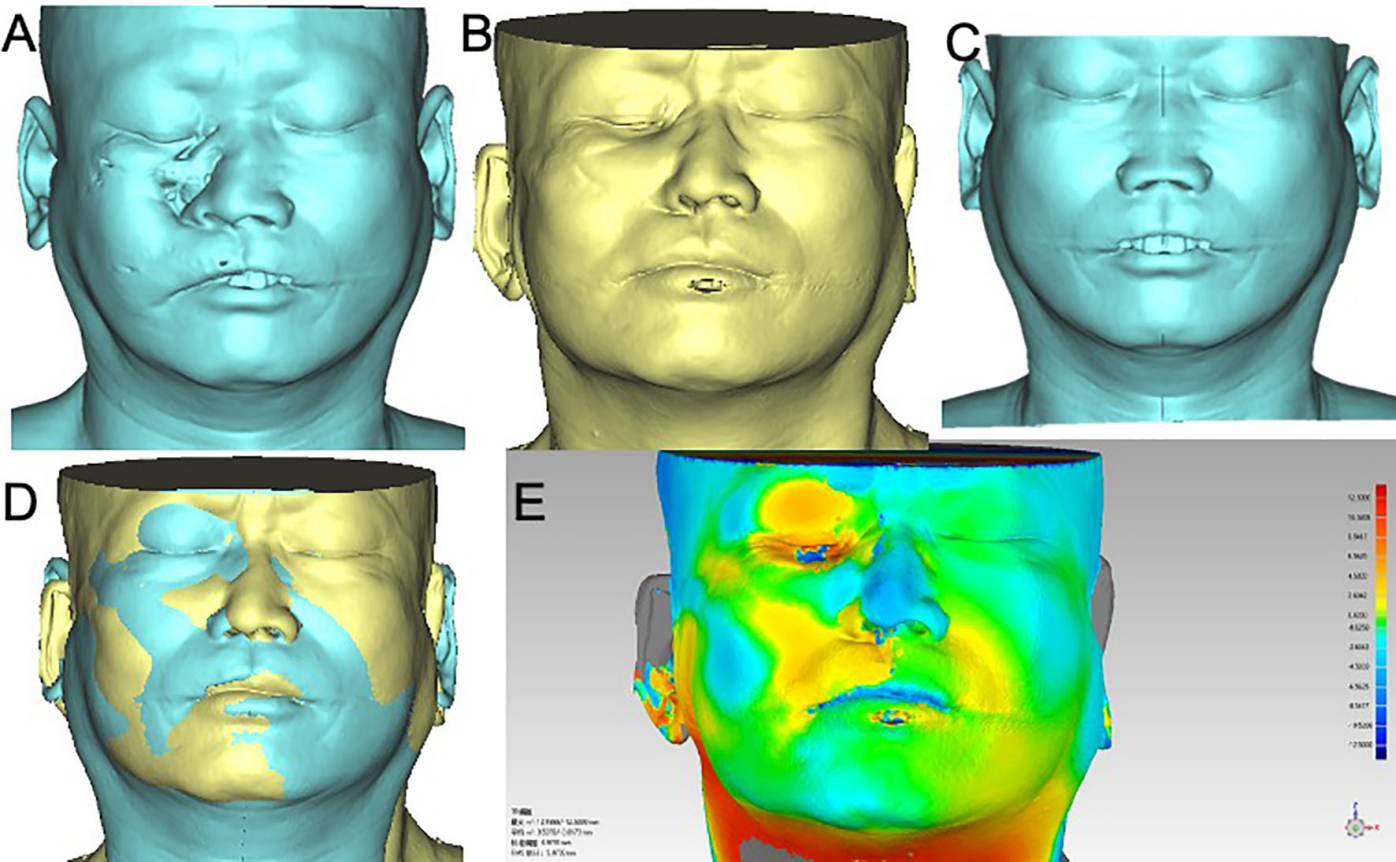
Soft tissue contour of pre-operation and post-operation alignment in FHS group. **(A)** Pre-operation soft tissue contour; **(B)** post-operation soft tissue contour; **(C)** mirrored hemiface of normal side to form a symmetrical face; **(D)** post-operation soft tissue contour aligned with the symmetrical face in **(C)**; **(E)** the color-gradient map indicating the differences of soft tissue contour in **(D)**, red color indicates ridge area, and blue indicates depressed area.

**Figure 7:**
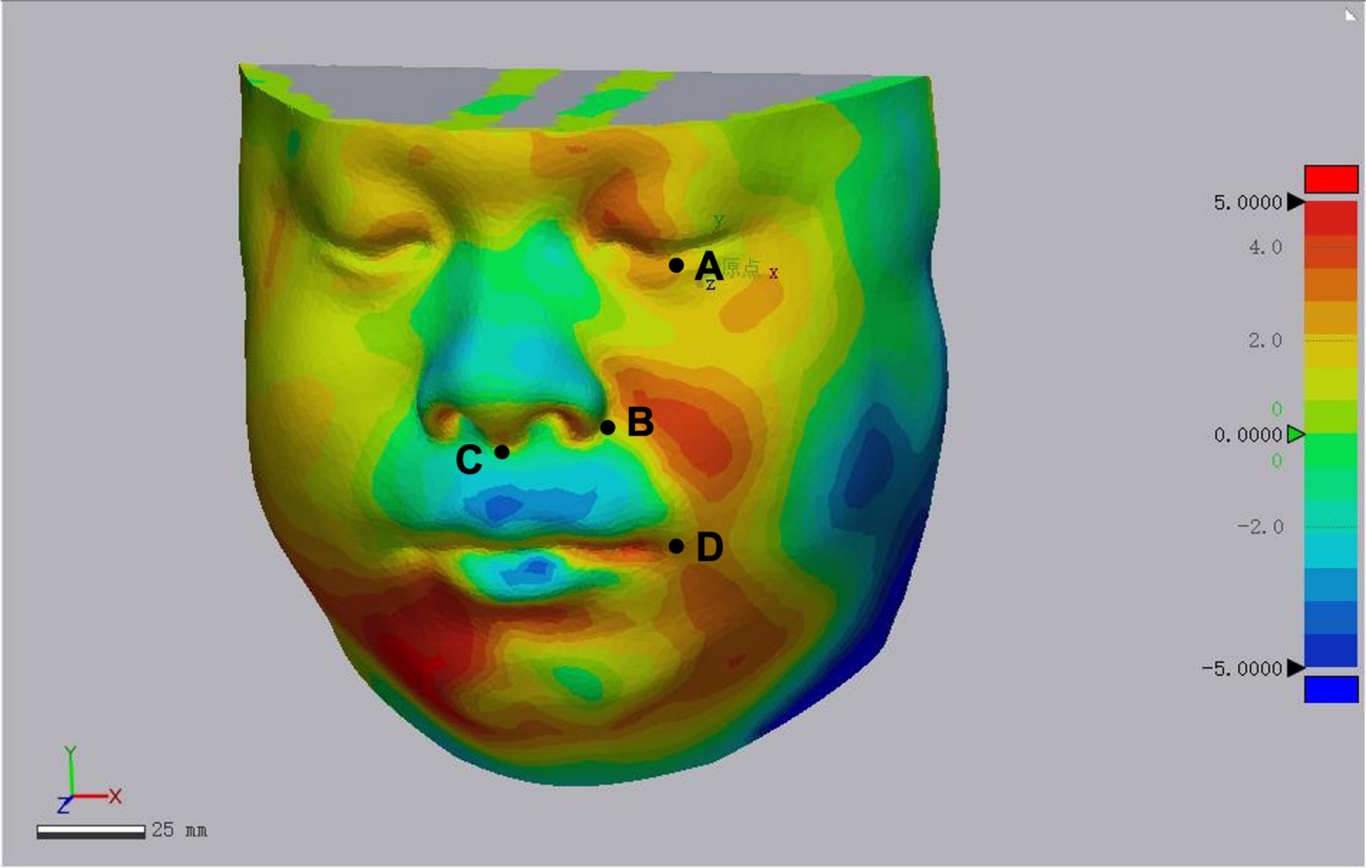
Quantitative analysis of soft tissue appearance of reconstructed face. **(A)** Point 1: midpoint of lower eyelid; **(B)** point 2: the cross point of ala nasi and nasofacial sulcus; **(C)** point 3: the point of philtrum; **(D)** point 4: point of mouth corner.

Postoperative soft tissue contour of all case was analyzed using Geomagic studio software (Raindrop, USA) (**Figures 5**, **6**). Subsequently, a mask was established using soft tissue thresholds, and a 3D model was generated by calculation. Using the mirror tool, a symmetrical face was obtained by mirroring to the normal hemiface. The.STL files of the symmetrical face and postoperative face exhibiting the soft tissue contour were imported into the software of Geomagic studio. The 3D models of symmetrical face and postoperative face were aligned with some anatomy landmarks, including nose, supraorbital and infraorbital line, zygoma, and midline. Then a color-gradient map showing differences in soft tissue contour of the reconstructed face and the contralateral normal side was obtained. The color-gradient maps were quantitatively analyzed using Geomagic control X (3D systems, USA). Four key points were set to analyze: Point1: midpoint of lower eyelid; Point 2: the cross point of ala nasi and nasofacial sulcus; Point 3: the point of philtrum; Point 4: point of mouth corner (**Figure 7**). The location of every point on the reconstructed face and the mirrored face was recorded as a three-dimensional coordinate, and the shift of every point was analyzed in X, Y, and Z axis, respectively. SPSS (Statistic Package for Social Sciences) 13.0 was used for data analysis. Two-tailed t-test was used for statistical analysis.

## Results

### Patient Demography

In the VSP group, there are 14 male and 6 female patients with the average age of 37.2 years (range from 12 to 52 years). Of the 20 subjects, 7 patients underwent primary reconstruction and 13 patients underwent secondary maxillary reconstruction. According to Brown’s revised defect classification of maxilla and midface, cases in this study were classified upon vertical defect or horizontal defect. Eight cases had undergone radiation therapy at the time of reconstructive surgery. To reconstruct the defect, 17 cases were designed using fibula flap, and in the other cases, ICFF were carried out. During follow-up, seven patients got implant-anchored prosthesis.

In the FHS group, there are nine male and five female patients with the average age of 37.9 years (range from 25 to 58 years). Half of the patients underwent primary reconstruction, and the other half underwent secondary maxillary reconstruction. Among them, three patients had received radiotherapy at the time of reconstructive surgery. The vertical and horizontal defect was classified as described above. All cases of defect were repaired with fibula flap.

Comparison of perioperative characteristics including fibula segments, operation time, blood loss and transfusion, patient cost is shown in **Supplementary Table 1**. Two-tailed t-test was used for statistical analysis and showed no statistical significance between two groups.

### Complications

In VSP group, titanium mesh was used in four cases to establish the floor of orbit and the infraorbital rim for class III vertical defect, while two cases, belonging to secondary maxillary reconstruction, exhibited exposure of the titanium mesh. Additional operation was performed to remove the mesh, and then enophthalmos developed. Temporalis muscle or free fat tissue transplantation was performed to improve the height of globe. The infraorbital rim was not settled up in only one case showing enophthalmos. In the VSP group, flap vascular crisis occurred in three cases, and all flaps were rescued.

In the FHS group, titanium mesh was used in three cases and exposed in two cases of class III vertical defect. In one case of class IIId (**Figure 3B**), the anterior part of mandibular ramus including coracoid was cut off from sigmoid notch to retromolar area, with attachment of temporalis muscle. This pedicled bone graft was shaped and placed to form the floor of orbit, and enophthalmos was prevented, while infraorbital rim was depressed yet. In the FHS group, flap vascular crisis occurred in two cases, and one of them was not rescued.

### Reconstructive Accuracy Analysis

The bone and soft tissue contour of all patients were established and classified according to the vertical defect classification, and the corresponding primary or secondary reconstruction was shown in **Figures 2**, **3**. For the aesthetical assessment, color-gradient map indicating the soft tissue contour difference was performed in cases of both VSP and FHS group. The typical cases of two groups are exhibited in **Figures 5**, **6**, respectively. In case of FHS group, the differences in area of infraorbital rim and the maxillary alveolar ridge were much bigger than that of case in VSP group, exhibiting larger area of orange and blue. In the VSP group, **Figure 2A** shows that a case of vertical class II reconstructed with ICFF exhibited a uniform change in the infraorbital region, and **Figure 2B** shows that a case of vertical class III reconstructed with fibula flap and titanium mesh exhibited better contour of infraorbital rim than the cases without titanium mesh as shown in **Figures 2D** and **3B**. For secondary reconstruction, cases in VSP group had smoother curve in the reconstructed region, whereas cases in FHS group showed some point with deep blue indicating obviously depressed surface. In vertical class III cases, the differences in orbit height (ΔD) and orbit volume (ΔV) between normal side and reconstructed side were measured and analyzed by t-test, the result of which is listed in **Table 2**. Mean ΔD is 1.78 ± 1.33 mm in VSP group and 4.25 ± 0.95 mm in FHS group, while mean ΔV is 2.04 ± 0.85 cm^3^ in VSP group and 3.25 ± 0.17 cm^3^ in FHS group. The differences in VSP group were significantly smaller than that in FHS group (**Figures 4D, E**).

**Table 2:** Orbit height and orbital volume analysis in VSP and FHS group.

Group	D1(mm)	D2(mm)	ΔD=|D1−D2|(mm)	ΔD(mean ± SD)	V1(cm^3^)	V2(cm^3^)	ΔV=|V1−V2|(cm^3^)	ΔV(mean ± SD)
VSP Group	35.9	40	4.1	1.78 ± 1.33	29.1	31.7	2.6	2.04 ± 0.85
	39.5	38.7	0.8		31.7	29.3	2.4	
	35.9	37.2	1		31.1	30.2	0.9	
	39.5	34	1.6		36.9	35.5	1.4	
	37.2	36.2	1.4		23.4	20.5	2.9	
FHS Group	39	43.2	4.2	4.25 ± 0.95	30.7	34.3	3.6	3.625 ± 0.17
	34.2	39.8	5.6		31.2	34.6	3.4	
	35.7	37.4	3.7		38	34.2	3.8	
	39.1	42.6	3.5		35.1	31.4	3.7	

The results of quantitative analysis of soft tissue appearance of reconstructed face are shown in **Table 3**. The distance shift in Z axis of Point 1 (midpoint of lower eyelid) and Point 4 (point of mouth corner) had statistical significance indicating more accurate of the reconstructed appearance.

**Table 3:** Quantitative analysis of soft tissue appearance of reconstructed face.

Point	Point shift distance (mm, mean ± SD)			P-value
	Axis	VSP	FHS	
Point1	X	0.3232 ± 0.3474	0.6635 ± 0.3167	0.0577
	Y	0.2646 ± 0.3392	0.4176 ± 0.4272	0.4103
	Z	1.2363 ± 0.8999	3.5575 ± 1.7475	**0.0012**
Point2	X	0.7241 ± 0.6503	0.4945 ± 0.4507	0.4477
	Y	0.3079 ± 0.2923	0.1984 ± 0.2720	0.4491
	Z	1.3739 ± 0.5251	1.5446 ± 1.5356	0.7181
Point3	X	0.0966 ± 0.0841	0.1298 ± 0.1868	0.5933
	Y	0.4563 ± 0.5003	0.3025 ± 0.2453	0.4892
	Z	0.981 ± 0.8027	0.5656 ± 0.6026	0.2770
Point4	X	1.2703 ± 0.7117	1.6929 ± 2.4254	0.5613
	Y	1.2482 ± 0.7419	1.5368 ± 1.1510	0.5164
	Z	1.5765 ± 0.7210	3.0753 ± 2.5003	**0.0340**

Significant P values bolded.

## Discussion

The maxillectomy usually produces complex defect involving several structures, like tooth-bearing alveolar, nasal cavity, orbit cavity, palate, etc. Bone reconstruction was recommended for the defect higher than class II according to the Brown’s vertical classification, while class I vertical defect was usually repaired by prosthesis (14). The complexity of maxillary reconstruction lends itself well to VSP surgery, which streamlines the shape of bone graft and improves the surgical and anatomical accuracy.

In VSP surgery, with the mirror function of the software, the images of contralateral normal maxilla were superimposed over the asymmetric flap side. The virtual plan and the bony framework were determined according to the mirrored normal maxilla to achieve a symmetric contour. With the introduction of 3D printing models and fabrication of surgical guides, complex resections can be planned preoperatively. In the reconstruction process, custom-made cutting guides helped to decide location and angulation of osteotomies, and the reposition guides determined precise position of the bone graft conducive for prosthesis rehabilitation. Studies analyzed vertical and horizontal changes between bone graft and normal side had demonstrated the better accuracy of VSP than FHS or traditional surgery (16–19). In our study, similar conclusion was obtained by comparing the orbit height and orbit volume in vertical defect Class III cases. More similar of orbit height and orbit volume in the reconstructed side indicated better sight function. And as shown in **Table 1**, seven cases in VSP group got implant denture, while no one in FHS group got prosthesis rehabilitation.

Complication comparation between two groups revealed a higher rate of complication in FHS groups (10/14). The other comparison of operation time, blood loss and transfusion, patient cost revealed no statistical difference in two groups, too, indicating that application of virtual surgical plan and 3D print in maxillary reconstruction was safe and economical and acceptable with better result.

To achieve the ideal goal of maxillary reconstruction, several variants of forearm flap, anterolateral thigh flap, fibula flap, scapular flap, and iliac crest free flap had been described. The fibular flap may be harvested as osseous flap, or chemical flap with muscle or muscle and skin paddle allowing to repair multi-kind of potential defects. And fibula flap usually provides a bone graft longer than 20 cm, which can be osteotomized into multiple segments (20, 21). Proper osteotomy makes the curve of fibula bone graft align well to the alveolar arch and maxillary buttress. The drawback of fibula flap was obvious that the width of the bone, 10 to 15 mm, was not enough for high maxillectomy defect. For class III vertical defects, titanium mesh or artificial-like specified PEEK should be installed combined with fibula bone graft. However, in secondary reconstruction, especially in cases with a history of radiation therapy, exposure of titanium mesh or artificial happened frequently. In our research, exposure of titanium mesh or PEEK occurred in three cases of VSP group and two cases of FHS group. In this situation, soft tissue like fat tissue of the skin paddle or flexor pollicis longus muscle is needed to insert between the recipient skin and titanium mesh. Alternatively, double barrel of fibula bone graft can be taken into consideration. Just as shown in **Figure 3D**, the infraorbital rim and alveolar arch were both restored by bone tissue, exhibiting a good contour of bony framework. But the soft tissue contour showed a depressed area between this two-layer bone graft, which reduced aesthetics. The ICFF comes to be a better choice.

The ICFF, as described by Taylor, Sanders, and Mayou (22, 23), has demonstrated considerable utility in midface reconstruction when used as either an osseous, osteocutaneous, or osteomyocutaneous composition. ICFF provides a bulky bone graft of about 9 cm in length, which is enough for a hemimaxilla reconstruction, and 4 cm in height, which is enough for repairing a class III vertical defect. The iliac crest bone graft can be shaped to conform to the contour of infraorbital rim. Otherwise, the combination of ICFF with titanium mesh, if needed, prevents the exposure of titanium mesh. The iliac crest bone graft was a whole piece in vertical, which is another advantage of ICFF, preventing the depressed soft tissue contour as mentioned before in **Figure 3D**. In the situation of bulky soft tissue defect, ICFF can be raised with the internal oblique, assisting line oral cavity and nasal cavity wound. Furthermore, the caliber of the vessel pedicle of ICFF, the deep iliac circumflex artery (DCIA), is consistent with superficial temporal artery. Thus, adoption of ICFF for maxillary reconstruction should be considered and popularized in clinical practice.

Numerous researches before focused on the bone framework and the accuracy of bone graft position in VSP surgery, and the advantage of virtual surgery planning in modeling bone framework was obvious, but in terms of appearance or soft tissue contour, VSP was not always that useful. In our research we reported the soft tissue contour analysis and suggested that “Bone is bone, soft tissue is independent”. To achieve aesthetical results, more attention should be given to soft tissue reconstruction, especially in some secondary reconstruction cases. For secondary reconstruction, the soft tissue defect is usually more complex than bone defects because of scar contracture deformities or stiffness caused by radiation therapy, limiting the applicability of VSP in real clinical practice. In our study, the exposure and inflammation of titanium mesh was not rare in VSP group. Titanium mesh was used in four cases, and two cases of secondary reconstruction exhibited titanium mesh exposure (**Table 1**). For secondary cases of class III vertical defect, soft tissue surrounding infraorbital region collapsed at varying degrees. When the contracture tissue was released completely with Weber-Fergusson incision, titanium mesh increased tension of the suture. The artificial infraorbital rim shaped by the titanium mesh overlapped with the incision, increasing the possibility of titanium mesh exposure greatly. Therefore, additional soft tissue was needed to cover the mesh, especially in cases after radiation therapy. In the case of complications of the exposed mesh, a secondary operation was performed to remove the mesh while transferring fat or pedicled muscle to prevent enophthalmos. The strategy that combines one-layer fibular bone graft and titanium mesh had been abandoned for reconstruction of class III vertical defect. Butterwort and Rogers reported a method of zygomatic implant perforated microvascular soft tissue flap (ZIP) to repair maxillary defect (24). For cases with bulky soft tissue deficit, using ZIP to fill the defect with soft tissue and rehabilitate occlusion by zygomatic implants, improved the quality of life, which could be taken into consideration.

Our research retrospectively analyzed 34 cases of maxillary reconstruction and proved the safety, accuracy, and functionality of virtual surgery *versus* traditional free-hand surgery. However, limitations remain in the application and the ultimate outcomes. For example, the application of virtual surgical plan and custom-made cutting guide simplified the bone framework modeling and positioned the bone graft precisely, but errors still occurred at the time of titanium plate shaping and fixation, for example. In this situation, introducing a navigation system that can discover the errors during surgery contributes to improve the outcomes. Also, we focused on the soft tissue appearance after surgery and revealed that soft tissue contour was an independent topic and should be taken into consideration. As the development of surgery planning software, we expect the utilization of soft tissue planning in software. Further study in maxillary reconstruction could focus on occlusion rehabilitation, combination of virtual surgical plan and navigation, aesthetical soft tissue reconstruction, etc., to resolve the limitation of this research.

## Conclusion

VSP surgery and 3D printing techniques helped to improve accuracy and precise rehabilitation of normal contour, especially in bone framework. But soft tissue appearance was not always reflected by the bone contour, suggesting flexible treatment strategies to suit particular circumstance. In addition, the virtual planning of soft tissue reconstruction deserves further investigation.

## Data Availability Statement

The original contributions presented in the study are included in the article/**Supplementary Material**. Further inquiries can be directed to the corresponding author.

## Ethics Statement

The studies involving human participants were reviewed and approved by the Ethics Committee of Shanghai Ninth People’s Hospital, Shanghai Jiao Tong University School of Medicine. Written informed consent from the participants’ legal guardian/next of kin was not required to participate in this study in accordance with the national legislation and the institutional requirements. Written informed consent was obtained from the individual(s) for the publication of any potentially identifiable images or data included in this article.

## Author Contributions

YW drafted the manuscript. XQ and JJ analyzed data. JS and CZ revised the paper. YH conceived the study design. All authors contributed to the article and approved the submitted version.

## Funding

This work was supported by the Shanghai Natural Science Foundation of China (grant numbers 17ZR1416300, 21ZR1453900), Shanghai Municipal Education Commission Gaofeng Clinical Medicine Grant Support (20152222), Fundamental research program funding of Ninth People’s Hospital affiliated to Shanghai Jiao Tong University School of Medicine (JYZZ146).

## Conflict of Interest

The authors declare that the research was conducted in the absence of any commercial or financial relationships that could be construed as a potential conflict of interest.

## Publisher’s Note

All claims expressed in this article are solely those of the authors and do not necessarily represent those of their affiliated organizations, or those of the publisher, the editors and the reviewers. Any product that may be evaluated in this article, or claim that may be made by its manufacturer, is not guaranteed or endorsed by the publisher.

## Supplementary Material

Supplementary Table 1 Comparison of perioperative characteristics.
