# Supplementary material for: Aesthetical and Accuracy Outcomes of Reconstruction of Maxillary Defect by 3D Virtual Surgical Planning
Source: Front Oncol. 2021 Oct 19;11:718946. doi: 10.3389/fonc.2021.718946 (PMC8560731; doi:10.3389/fonc.2021.718946)
Supplement: Supplementary Table 1 — Comparison of perioperative characteristics. [file Table_1.pdf]

Table S1 Comparasion of perioperative characteristics

Perioperative characteristics	VSP Group	FHS Group	P-value
Fibula segments	2.64±0.63	2.86±0.77	0.1117
Operation time (hour, mean±SD)	8.75±2.37	8.56±3.25	0.8825
Blood loss（ml, mean±SD）	1070±795	822±377	0.4069
Blood transfusion（ml, mean±SD）	560±540	467±412	0.6800
Patient cost （dollar, mean±SD）	20595±7528	20878±4655	0.9214
